# The effect of a conflict of interest disclosure form using closed questions on the number of positive conflicts of interest declared – a controlled study

**DOI:** 10.7717/peerj.128

**Published:** 2013-08-13

**Authors:** Christopher Baethge

**Affiliations:** Deutsches Ärzteblatt and Deutsches Ärzteblatt International, Editorial Offices, Cologne, Germany; Department of Psychiatry and Psychotherapy, University of Cologne Medical School, Cologne, Germany

**Keywords:** Conflict of interest declarations, Conflict of interest forms, Conflict of interest statements, Conflicts of interest, Competing interests, Medical publishing, Journalology, Periodicals as topic, Deutsches Ärzteblatt International, ICMJE

## Abstract

**Objective.** While declarations of conflicts of interest (COI) have become an integral part of medical articles, COIs are often not declared completely and accurately. One of several possible reasons for deficient COI declarations is the lack of standardized and comprehensive COI forms. In 2010, the International Committee of Medical Journal Editors (ICMJE) introduced a COI form using clear definitions and closed questions. Deutsches Ärzteblatt (DA), the journal of the German Medical Association, adapted this form and implemented it in early 2011. However, it is unclear whether changing COI forms leads to more positive COI statements.

**Material and Methods.** In a controlled pre-post design, positive COI statements were analyzed at three German medical journals: one had changed its COI form (DA), two had not: Deutsche Medizinische Wochenschrift (DMW) and Nervenarzt (Ner), both of whom used open questions in their forms. At the levels of both authors and articles, respectively, the proportion of positive COI declarations in orignal and review articles was recorded for volumes 2010 (before implementation of the new COI form at DA) and 2012 (after). The change in positive COI disclosures at the journals was compared. Chi-square tests were used to compare the figures by journal in 2010 versus 2012 and among DA, DMW, and Ner.

**Results and Discussion.** While positive COI statements more than doubled at DA, there was no meaningful change in either of the control journals: In 2010, 19.1% [95% CI: 15.4–23.2] of all DA-authors submitted positive COI declarations, relative to 39.6% [35.0–44.5] in 2012, a factor of 2.1. At the level of articles, positive COI statements increased from 32.3% [23.7–42.0] to 70.1% [60.5–78.6] (factor 2.2). At DMW, positive declarations rose by a factor of 1.3 to 12.1% [9.7–14.8] in 2012 at author level and by a factor of 1.3 to 19.4 [14.2–25.7] for articles. At Ner, figures fell: to 19.9% for authors [16.9–23.4] and 30.7% for articles [24.0–38.1] (both by a factor of 0.8). *P*-values for the comparison of positive COI statements between 2010 and 2012 were low at DA (*p* for both author and article level comparisons <0.00001) and considerably higher at DMW and Ner (all >0.05). Although this is not a randomized controlled study, the findings support the hypothesis that the steep increase in positive COI statements at DA from 2010 to 2012 is the result of its new COI form: Relative to two journals that had not modified their COI forms the effect size of the change was considerably higher at DA. Also, in contrast to DMW and Ner, *p*-values indicate that chance is unlikely to have played a major role in the change of positive COI statements at DA.

**Conclusions.** COI forms employing closed questions based on clear definitions of conflicts of interests, such as those recommended by ICMJE and now used by Deutsches Ärzteblatt, seem to be superior to less structured forms. These results require confirmation with other COI forms and at other journals.

## Introduction

Since a landmark study in 1998 (Stelfox et al., 1998) found a positive association between authors’ cooperation with pharmaceutical companies producing calcium-channel antagonists and article content favourable towards this class of drugs, a host of articles have confirmed the importance of conflicts of interest in medicine (for reviews see Lexchin et al., 2003; Bekelman, Li & Gross, 2003; Schott et al., 2010a; Schott et al., 2010b; Lundh et al., 2012). As a result, it is the current understanding that readers of scientific articles should be aware of conflicts of interest (COI) so that they can judge a paper in a more informed way. With scientific papers, therefore, many medical journals publish the authors’ conflicts of interest (Ancker & Flanagin, 2007; Baethge, 2011). However, an underreporting of conflicts of interest has been observed in several studies (Okike et al., 2009; Chimonas, Frosch & Rothman, 2011; Kesselheim etal, 2012).

What authors declare as a conflict of interest may depend on a variety of factors, including the manner in which declarations of competing interests are requested by journals. For example, at Deutsches Ärzteblatt (DA), the journal of the German Medical Association, before 2005, the instructions for authors requested authors to state their conflicts of interest but there was no obligation for authors to submit a written declaration. When a written and signed COI statement was introduced in 2005, the proportion of positive COI declarations increased from zero to about 30% of articles (Baethge, 2008). This statement was accompanied by the definition of conflicts of interests by the International Committee of Medical Journal Editors (ICMJE), together with pertinent examples and a request that any such competing interest be stated (see Appendix S1). However, this request was posed in an open fashion: “Please state all conflicts of interest as defined above”.

In late 2010 the ICMJE published an updated version of their COI form (Drazen et al., 2010; ICMJE, 2010). One of the major advantages of this new format is that it consists of closed questions. For example, it asked after a short introduction, “Are there any relevant conflicts of interest?” The author then has to check a box (yes/no) and to state the concrete COI. In addition, the new disclosure form covers a broad range of possible conflicts of interest on both personal and institutional levels such as grants, patents, and also non-financial conflicts of interest.

Inspired by and based on the ICMJE disclosure form, Deutsches Ärzteblatt revised its own form (Lieb et al., 2011). The form now consists of seven domains, e.g., payment related to education and conferences, and a set of 16 closed questions, e.g., “Have you received payment for a lecture or for preparing scientific or educational events connected to this topic?” If this is answered in the affirmative, competing interests have to be specified (Appendix S2).

However, while at Deutsches Ärzteblatt it was hypothesized that the new structure of the COI declaration would be clearer for authors and would better guide them in stating their competing interests, it was unclear whether changing the format of a written COI declaration changes the percentage of positive COI statements. Therefore, the rates of positive COI statements before and after initiation of the new COI form were analyzed and the results were compared to those of two journals whose COI forms remained unchanged.

## Material and Methods

This is a retrospective, controlled study of conflict of interest statements in three medical journals: Deutsches Ärzteblatt (DA), Deutsche Medizinische Wochenschrift (DMW), and Der Nervenarzt (Ner). The latter periodical is a specialty journal devoted to psychiatry and neurology; the other two journals are general medical journals. While all journals appear in German, Deutsches Ärzteblatt is also distributed in an international online edition in English, Deutsches Ärzteblatt International.

The control journals (DMW, Ner) were selected for this analysis because it was known that they did not change their COI policy over the period under study and were selected in order to include one general medical journal and one specialty journal. They also represent the two largest medical publishers in Germany: Springer and Thieme. There are only a few scientific general medical journals in Germany, and only DMW is distributed weekly – like Deutsches Ärzteblatt. Therefore, DMW was selected as a control. Der Nervenarzt was included because it was considered representative for specialty journals in German. It is published by scientific associations (the associations of Neurology and Psychiatry), appears monthly, and publishes many review articles. Also, in a recent study it had been identified as one of the few general psychiatry journals in Germany that published COI statements at all (Heidenreich & Baethge, 2012). For feasibility, the number of comparison journals was kept to two. All original and review articles in the three sources are accompanied by conflict of interest statements. Both types of articles were included because an association between the competing interests of the authors and the content of their articles has been shown for both original research articles (for example, Friedmann & Richter, 2004) and reviews (for example, Barnes & Bero, 1998). Also, review articles play a major role in all the journals under study.

In 2010, all three journals’ COI forms used an open question approach. Of the three journals, only Deutsches Ärzteblatt adopted a revised version of ICMJE’s new COI statement proposed in 2010 (Appendix S2). This brought about a major change for Deutsches Ärzteblatt, because the format was changed from one open to several closed questions.

While Deutsches Ärzteblatt’s form is clearly based on the updated ICMJE form, there are some modifications: These include a regrouping of the questions and a different time period covered by the questionnaire. At Deutsches Ärzteblatt, the time period is five years, whereas in ICMJE’s form it is “the duration of the project at issue” and three years for all relevant conflicts of interest outside the submitted work, that is, outside the “project at issue”. Also, in its section on relevant financial relationships outside the submitted work, the ICMJE form inquires about relationships “that could be perceived to influence, or that give the appearance of potentially influencing, what you wrote […]”. In contrast, Deutsches Ärzteblatt’s form asks for relationships that exist or have existed with parties who could have an interest in the manuscript at hand – without differentiating between funding for a concrete study and financial activities outside the submitted work. However, the most important characteristic of ICMJE’s new COI statement, a closed question format, was retained. Deutsches Ärzteblatt implemented its new COI form in February 2011 (Lieb et al., 2011). Hence, for articles published in 2011, two different COI forms were used depending on the date of submission. As a result, this study focuses on the analysis of articles published in 2012, by which time all authors were using the new COI form.

DMW requires a written and signed statement from each author regarding financial cooperation with companies whose products (or competitor’s products) play a major role in the article (DMW, 2008). The COI declaration of Nervenarzt (Nervenarzt, 2013) is similar to that used by Deutsches Ärzteblatt between 2005 and 2011 insofar as it defines conflicts of interests in the sense of the Uniform Requirements for Manuscripts Submitted to Biomedical Journals. Nervenarzt’s COI form differs, however, in that it asks the corresponding author to report on conflicts of interests for all authors.

In order to investigate the effect of the new COI form at Deutsches Ärzteblatt, all original and research articles published in 2010 (before implementation) and 2012 (after implementation) were analyzed. The proportions of authors and articles with positive COI statements were compared. As controls, all original and review articles in DMW and Nervenarzt published in 2010 and 2012 were analyzed in the same way.

The results are presented as raw numbers, percentages and 95% confidence intervals. Chi-square tests were used to compare proportions of positive COI statements before and after implementation of the new COI form at Deutsches Ärzteblatt and to compare positive COI form proportions among the three journals – both at the author and article levels. In the absence of earlier studies on the effect of COI forms on the proportion of positive competing interest statements, effect sizes are given to add another level of evaluating the comparisons. As a measure of effect sizes, standardized mean differences are presented (chi-square values were transformed into Cohen’s d) because they are more established in medicine than more abstract measures, such as Cramer’s V. Because this study is not randomized, probability values are given but are not labeled significant or non-significant.

## Results and Discussion

### Results

The proportion of positive COI statements doubled at Deutsches Ärzteblatt between 2010 and 2012: from 19.1% to 39.6% at the author level (factor 2.07) and from 32.3% to 70.1% at the article level (factor 2.17). No such increase was observed at the two control journals: positive COI statements increased less pronouncedly at DMW (by a factor of 1.34 for authors and 1.24 for articles). At Nervenarzt the figures declined (by a factor of 0.82 and 0.78 for authors and articles, respectively). Table 1 presents the data in detail, including 95% confidence intervals.

**Table 1:** Positive COI statements in 2010 compared to 2012 at the author and article levels in three German journals. Presented are percentages (bold), 95% confidence intervals (in square brackets), and raw number ratios (*n*/*N*).

		Deutsches Ärzteblatt	Deutsche Medizinische Wochenschrift	Nervenarzt
**Authors**	2010	**19.1** [15.4–23.2] 74/388	**9.0** [7.0–11.4] 59/656	**24.4** [20.7–28.6] 111/454
	2012	**39.6** [35.0–44.5] 159/401	**12.1** [9.7–14.8] 75/622	**19.9** [16.9–23.4] 119/597
**Articles**	2010	**32.3** [23.7–42.0] 32/99	**15.7** [10.7–21.8] 26/166	**39.4** [31.5–47.8] 54/137
	2012	**70.1** [60.5–78.6] 68/97	**19.4** [14.2–25.7] 35/180	**30.7** [24.0–38.1] 50/163

**Figure 1:**
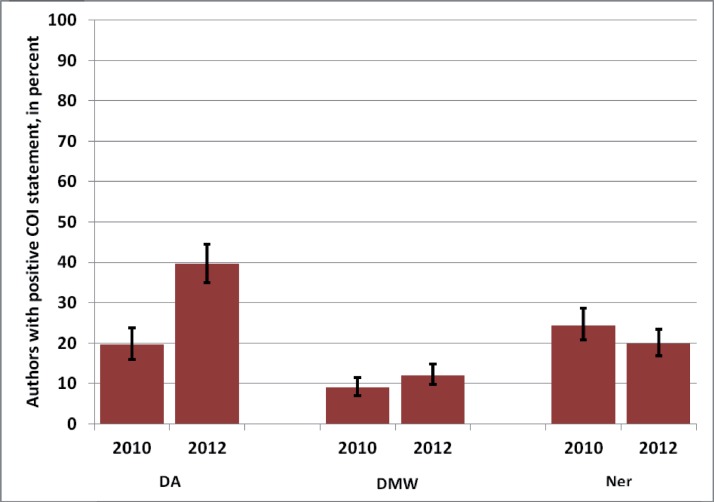
Percentage of authors of published articles with positive COI statements in 2010 versus 2012 in three German journals. DA, Deutsches Ärzteblatt; DMW, Deutsche Medizinische Wochenschrift; Ner, Der Nervenarzt. Whiskers indicate 95% confidence intervals.

**Figure 2:**
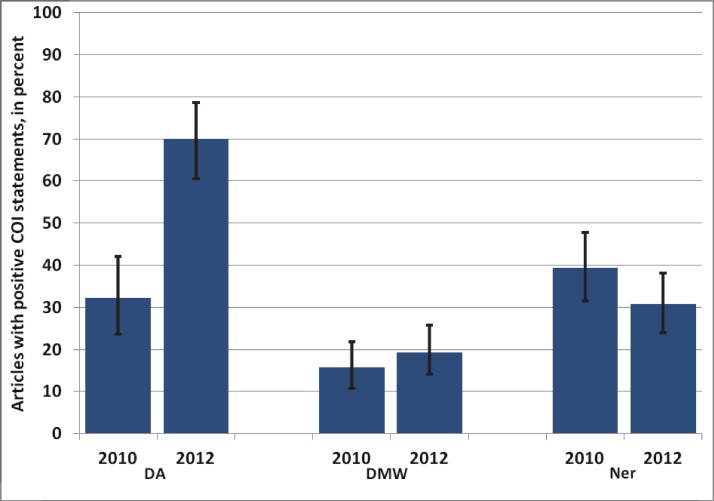
Percentage of articles with positive COI statements in 2010 versus 2012 in three German journals. DA, Deutsches Ärzteblatt; DMW, Deutsche Medizinische Wochenschrift; Ner, Der Nervenarzt. Whiskers indicate 95% confidence intervals.

The proportion of positive COI declarations was therefore more similar among the three journals in 2010 than it was in 2012, as shown in Figs. 1 and 2.

The effect sizes for the proportional differences in positive COI declarations between 2010 and 2012 at Deutsches Ärzteblatt were medium to strong at both author and journal levels (Cohen’s d 0.5 and 0.8, resp.). The corresponding figures for the two comparison journals, however, indicated low effect sizes (ranging from −0.2 to 0.1). The *p*-values for the comparison of the proportions of positive COI statements at Deutsches Ärzteblatt were low (*p* < 0.00001 at both the author and article levels), suggesting that chance is an unlikely explanation for the differences. However, since neither the differences observed at DMW nor the differences observed at Nervenarzt corresponded to low *p*-values (*p* = 0.074 and 0.356 for authors and articles at DMW, resp.; *p* = 0.079 and 0.113 for Nervenarzt), chance cannot be rejected as an explanation for the minor changes observed at those two journals. Table 2 documents the effect sizes and *p*-values for all comparisons.

**Table 2:** Effect sizes and *p*-values of comparisons. Absent prior experiences with effects sizes in this field conventions for the interpretation of standardized mean differences (Cohen’s d) are: 0.2 (weak effect), 0.5 (medium effect), 0.8 (strong effect). Cohen’s d was approximated from χ^2^ and *N* (df:1). Bonferroni correction for multiple testing (assuming 18 comparisons) results in an adjusted *p*-value of 0.0028.

Comparison	Effect size (Cohen’s d)	*p*-value
**Authors**		
DA 12 v DA 10	0.46	<0.00001
DMW 12 v DMW 10	0.10	0.074
Ner 12 v Ner 10	−0.11	0.079
		
DA 10 v DMW 10	0.30	<0.00001
DA 10 v Ner 10	0.13	0.060
DMW 10 v Ner 10	0.43	<0.00001
		
DA 12 v DMW 12	0.68	<0.00001
DA 12 v Ner 12	0.44	<0.00001
DMW 12 ver Ner 12	0.11	0.00017
**Articles**		
DA 12 v DA 10	0.82	<0.00001
DMW 12 v DMW 10	0.10	0.356
Ner 12 v Ner 10	−0.18	0.113
		
DA 10 v DMW 10	0.40	0.002
DA 10 v Ner 10	0.15	0.264
DMW 10 v Ner 10	0.56	<0.00001
		
DA 12 v DMW 12	1.15	<0.00001
DA 12 v Ner 12	0.83	<0.00001
DMW 12 ver Ner 12	0.26	0.016

## Discussion

This study yielded two findings. Firstly, a steep increase in positive COI declarations was found at Deutsches Ärzteblatt after a COI form employing closed questions had been introduced. This COI form was adapted from ICMJE’s new COI declaration statement. Secondly, no such increase was found in the two control journals that had not changed their COI policy.

This is a controlled pre-post study, not a randomized controlled trial (RCT); thus, the changes observed cannot be ascribed with certainty to the different COI forms used by the journals under study. However, desirable as RCTs are, at Deutsches Ärzteblatt the editorial staff felt that the disadvantages of using different COI forms in the same time period – meaning that different COI declarations published would mean different things to the readers within the same time period – outweighed the potential benefits of a randomized design. A randomized design would have impaired rather than improved transparency for readers. Even the randomized assignment of different COI forms to different journals is problematic. It is not clear how other characteristics of those journals, such as scope or different levels of editorial vigilance or experience, might affect the results. A large number of journals would be necessary to level out these confounders.

To the best knowledge of the author, no major changes took place at the journals under study in terms of scope, editorial staff, publishers, or the medical societies to which the journals belonged. However, while it is believed that the comparison journals selected in this study represent the majority of German language scientific medical periodicals, two journals are not enough to *prove* representativeness.

A two-fold increase is a large effect with a standardized mean difference of 0.8 at the article level (Cohen’s d). Also, the probability that the results are due to random variation is low, with exact *p*-values for the change at Deutsches Ärzteblatt of 2 × 10^−10^ (author level) and 1.2 × 10^−7^ (article level). Both of these formal factors support the conclusion that the differences observed from 2010 to 2012 are attributable to the change in COI forms.

Finally, there is a high *a priori* probability that modifying the COI form from open to closed questions would lead to different results. While open questions are superior where attitudes or opinions are of interest, closed questions are more likely to produce answers than open questions if specific factual information is sought (Schuman & Scott, 1987). Questionnaires regarding competing interests, however, focus on facts. Taken together, it is likely that the increase in positive COI statements is a consequence of the changed COI form in Deutsches Ärzteblatt.

The results of the present study support the hypothesis that using ICMJE’s new uniform COI declaration will result in more positive COI statements. And yet, the differences between ICMJE’s form and Deutsches Ärzteblatt’s form have to be noted: ICMJE distinguishes between funding for the present study and “relevant financial activities outside the submitted work”. At Deutsches Ärzteblatt, large sponsored trials are the exception, and smaller studies or review articles are the rule. As a result, many of the projects presented in the manuscripts submitted to Deutsches Ärzteblatt are not sponsored by parties other than the employer of the authors. This fact reflects the differences between high impact factor, research oriented journals and periodicals with review articles and papers on smaller studies. For the purposes of Deutsches Ärzteblatt and in the interest of simplicity, therefore, two different sections – one for the study presented and another for “relevant activities outside the submitted work” as in the ICMJE form – seemed inappropriate. It is important to note, however, that this difference is a matter of form and not of content, because Deutsches Ärzteblatt’s COI form covers all kinds of conflicts mentioned in ICMJE’s form.

In the same vein, the use of two different time periods of reference specified in ICMJE’s form (“any duration” for the study presented versus 3 years for other “relevant activities”) seemed unnecessary at Deutsches Ärzteblatt. However, the reference period at Deutsches Ärzteblatt (five years) differed from both periods used in ICMJE’s COI statement. At Deutsches Ärzteblatt, the editors decided to be consistent with Deutsches Ärzteblatt’s earlier form (used 2005–2011) and retained the five year reference period. However, it has to be noted that, to the knowledge of the author, there are no empirical data to support the use of any specific time frame. Therefore, journals have to find a plausible compromise between a time period that can be considered long enough to cover important conflicts of interest and the limits of memory. This particularly applies to minor forms of collaborations, such as the payment of travel expenses or conference fees. It is also conceivable that the impact of COI diminishes over time. Admittedly, in the absence of guiding data, setting the time frame for a COI statement is not random but arbitrary to a certain extent.

In addition, ICMJE’s wording in the section on relevant financial activities outside the submitted work was not adopted by Deutsches Ärzteblatt. ICMJE’s declaration form states: “This section asks about financial relationships with entities in the bio-medical arena that could be perceived to influence, or that give the appearance of potentially influencing, what you wrote in the submitted work”. While this phrasing is probably intended to increase the sensitivity of the questionnaire, it was felt at Deutsches Ärzteblatt that it puts the author in an awkward position. It may be difficult to imagine what others might perceive as influential, and it may be tempting for some authors, in line with their own interests, to interpret the notion of “potential influence” conservatively. Therefore, Deutsches Ärzteblatt’s form specifically asks for certain conflicts of interests. While this may be more specific, it is less inclusive than ICMJE’s formulation.

It has been shown that COI statements are often inaccurate. Kesselheim etal and co-authors (2012) recently showed that among all articles written by authors with competing interests as traced from litigation documents, only 15% declared their conflicts of interests correctly. Other studies have shown better rates of correct disclosure, but still far below completeness (Okike et al., 2009; Chimonas, Frosch & Rothman, 2011). Several factors may be responsible for this incompleteness of COI statements. Authors may lack knowledge of what constitutes a conflict of interest, authors may forget or be oblivious to the existence of a conflict, or authors may simply lie to journals. By leaving less room for interpretation, new COI forms, such as the one proposed by ICMJE and the one under study here, have the potential to improve this situation. Given the variety of reasons for inaccurate or incomplete COI declarations, it is tempting to speculate that underreporting will continue even with better forms. It is possible, however, that political developments will improve the reporting of competing interests. One such development is the Physician Payments Sunshine Act in the USA (requiring manufacturers to report their financial relations to individual doctors) that may lead to more transparency and to less underreporting.

## Conclusions

In summary, these data support the notion that the characteristics of COI forms influence the declaration pattern of authors, with closed questions eliciting more positive declarations of conflicts of interest than open questions. ICMJE’s uniform COI declaration therefore appears to represent considerable progress in increasing transparency around conflict of interest declarations, but some journals, particularly those publishing predominantly review articles and small studies, may benefit from revising this form according to their own needs.

## Supplemental Information

10.7717/peerj.128/supp-1 Appendix S1 Deutsches Arzteblatt’s COI form 2005–2011 [English] This is the COI form used before the new form (reference Deutsches Arzteblatt 2011) was implemented.

10.7717/peerj.128/supp-2 Appendix S2 Deutsches Ärzteblatt’s COI form (new) as used from 2011 on
