# Supplementary material for: The effect of a conflict of interest disclosure form using closed questions on the number of positive conflicts of interest declared – a controlled study
Source: PeerJ. 2013 Aug 13;1:e128. doi: 10.7717/peerj.128 (PMC3746959; doi:10.7717/peerj.128)
Supplement: Appendix S2 [file peerj-01-128-s002.pdf]

Deutsches Ärzteblatt International

Conflict of interest statement

Dear authors,

Deutsches Ärzteblatt International publishes with all articles in the medical-scientific section of the journal a declaration about conflicts of interest. This transparency helps editors and readers. We ask each author to fill in a signed declaration. A conflict of interest does not in itself cast doubt on the scientific quality of a manuscript.

Explanatory notes

A conflict of interests in the sense of the Uniform Requirements for Manuscripts Submitted to Biomedical Journals of the ICMJE (International Committee of Medical Journal Editors) and the "German Working Group on Conflicts of Interest in Medicine" exists if an author has financial or personal ties to third parties whose interests may be affected positively or negatively by the manuscript's content. The presence or absence of a conflict of interest does not depend on whether or not an author feels that a financial or non-material relationship affects his or her judgment.

The questionnaire overleaf is based on the recommendations of the ICMJE and the "German Working Group on Conflicts of Interest in Medicine" relating to conflict of interest declaration. It relates to the current calendar year and the previous five years. If in doubt, authors should err on the side of declaring conflicts of interest. Please see overleaf for important background information concerning conflicts of interest.

Please send your declaration to:

Deutsches Ärzteblatt, Medizinisch-Wissenschaftliche Redaktion, Ottostr. 12, 50859 Cologne, Germany
fax: +49 (0)2234 7011140, email: medwiss@aerzteblatt.de

To the questionaire ▶

Deutsches Ärzteblatt International

Name of author: ______________________________

Title of article: ______________________________

____

The following questions relate only to the current manuscript, but defined broadly. For example, if the article pertains to the epidemiology of hypertension, you should declare all relationships with manufacturers of antihypertensive medication, even if that medication is not mentioned in the manuscript. On the other hand, we do not require you to declare relationships, such as the holding of shares, in companies which have no connection with the topic discussed.

Part 1 Relations with companies, patents and royalties

Do you hold shares in a company which has an interest in the messages contained in this manuscript?

☐ Yes If yes, please specify: ______________________________

____

☐ No

Do you have any personal connections with a company (for example a partner employed there) which has an interest in the messages contained in this manuscript?

☐ Yes If yes, please specify: ______________________________

____

☐ No

Do you hold any patents which are connected to the subject matter of this paper?

☐ Yes If yes, please specify: ______________________________

____

☐ No

Do you hold any licenses or royalty agreements connected to the subject matter of this paper?

☐ Yes If yes, please specify: ______________________________

____

☐ No

Part 2 Consultancy roles

Have you received payment or consultancy fees (for example membership of an advisory board or steering committee) with any connection to the subject matter of the manuscript?

☐ Yes If yes, please specify: ______________________________

____

☐ No

Part 3 Authorship payments

Have you received payment for authorship or co-authorship of a publication related to this paper´s topic?

☐ Yes If yes, please specify: ______________________________

____

☐ No

Have you received assistance in drafting the present manuscript or other work on similar themes?

☐ Yes If yes, please specify: ______________________________

____

☐ No

Have you received payment for peer reviewing material on this subject?

☐ Yes If yes, please specify: ______________________________

____

☐ No

Part 4 Payment related to education and conferences

Have you received payment to cover the cost of participation fees in a conference or educational event related to this topic?

☐ Yes If yes, please specify: ______________________________

____

☐ No

Have you received any travel or accommodation expenses in connection with this topic?

☐ Yes If yes, please specify: ______________________________

____

☐ No

Have you received payment for a lecture or for preparing scientific or educational events connected to this topic?

☐ Yes If yes, please specify: ______________________________

____

☐ No

Part 5 Payment for scientific activity

Have you received payment for carrying out clinical studies related to this topic?

a) I have personally received funding
b) My department or institution has received funding
c) I am responsible for such funding (e.g. as director of a clinic or other service provider)

☐ Yes If yes, please specify: ______________________________

____

☐ No

Have you received monies (including support in kind such as equipment or organizational support) for an event initiated by you which bears a relation to this topic?
a) I have personally received funding
b) My department or institution has received funding
c) I am responsible for such funding (e.g. as director of a clinic or other service provider)

☐ Yes If yes, please specify: ______________________________

____

☐ No

Part 6 Other

Do you have any non-material conflicts of interest in relation to this topic, for example personal or academic opinions or membership of particular schools of thought?

☐ Yes If yes, please specify: ______________________________

____

☐ No

Do you have any other competing interests?

☐ Yes If yes, please specify: ______________________________

____

☐ No

Part 7 Who is your employer?

____

______________________________ ______________________________

Place, Date **Signature**
